# Surgical corridors to foramen magnum meningiomas: a mini-review

**DOI:** 10.3389/fneur.2023.1228285

**Published:** 2023-07-17

**Authors:** Matias Baldoncini, Sabino Luzzi, Joao P. Almeida, William Omar Contreras-López, Emanuele La Corte, Edgar G. Ordóñez-Rubiano, Alvaro Campero

**Affiliations:** ^1^Laboratory of Microsurgical Neuroanatomy, Second Chair of Gross Anatomy, School of Medicine, University of Buenos Aires, Buenos Aires, Argentina; ^2^Department of Neurological Surgery, Hospital San Fernando, Buenos Aires, Argentina; ^3^Department of Clinical-Surgical, Diagnostic and Pediatric Sciences, University of Pavia, Pavia, Italy; ^4^Neurosurgery Unit, Department of Surgical Sciences, Fondazione IRCCS Policlinico San Matteo, Pavia, Italy; ^5^Department of Neurosurgery, Mayo Clinic, Jacksonville, FL, United States; ^6^Functional Neurosurgery, NEMOD International Neuromodulation Center, Clínica Foscal Internacional, UNAB University, Bucaramanga, Colombia; ^7^Department of Neurosurgery, Fondazione IRCCS Istituto Neurologico Carlo Besta, Milan, Italy; ^8^Department of Biomedical and Neuromotor Sciences (DIBINEM), Alma Mater Studiorum University of Bologna, Bologna, Italy; ^9^Department of Neurological Surgery, Fundación Universitaria de Ciencias de la Salud (FUCS), Hospital de San José – Sociedad de Cirugía de Bogotá, Bogotá, Colombia; ^10^Department of Neurological Surgery, Padilla Hospital, Tucumán, Argentina

**Keywords:** skull base, foramen magnum, far lateral, condyle, meningioma

## Abstract

Gross-total resection of foramen magnum meningiomas remains the gold standard of treatment and should be performed whenever possible. The transcondylar approach (and its variations) represents the most used approach for meningiomas located in the lateral or anterior borders of the foramen magnum. Endoscopic transclivus approaches represent a useful option in selected cases of anterior midline foramen magnum meningiomas, to be performed in centers with advanced experience in endoscopic skull base surgery, with the caveats of increased risk of postoperative cerebrospinal fluid leak. Alternatively, radiosurgery remains an option for well-selected cases, especially for the management of asymptomatic patients with small enlarging tumors. Advances in molecular profiling, as well as genetic analysis, may guide adjuvant treatment.

## 1. Introduction

Meningiomas account for 13%–26% of all intracranial tumors (1) and represent the most common type of benign intracranial tumor. Foramen magnum meningiomas (FMM) represent 0.3%–3.8% of intracranial meningiomas and arise from the dura of the craniovertebral junction (CVJ) (2–5). The unique location makes this tumor one of the most challenging in skull base surgery since it can arise at any location on the perimeter of the foramen magnum. FMM are classified according to their neurovascular relationships and anatomical extension (5). Ventral lesions are located in the inferior third of the clivus (basal groove) projecting to the superior edge of the C2 body anteriorly and present a slow growth pattern, generally leading to late symptomatology and a delayed diagnosis (5). Clinical symptoms are heterogenous and FMM can be misdiagnosed. Variable symptoms can be found including headache, cervical pain (unilateral/bilateral), sensory deficits, swallowing dysfunction, or even more severe symptoms in large tumors, including motor deficit progressing to quadriplegia. Clinical FMM triad is described as cold, clumsy hands with intrinsic hand atrophy (6). Tumor location (anterior, lateral, or posterior) defines the most reasonable approach for each case. Usually, most lateral and posterior FMM can be approached using the conventional inferior suboccipital approach (7). In contrast, ventral FMM often requires more complex approaches. Different techniques have been described to avoid injury of cranial nerves and posterior circulation arteries in relationship with the tumor.

Given the critical location of FMM and that complete resection of the basal dura is not usually feasible, a Simpson grade 2 is achieved in the majority of cases (8). Thus, the Simpson grade scale should be used carefully. However, the message of the Simpson grade scale of maximizing the extent of resection and minimizing morbidity remains the gold standard in all scenarios (9, 10). Unfortunately, as these tumors are infrequent and very difficult to treat, controversies on surgical approaches persist. This mini review provides the readers with resumed information on schools of thought in surgical corridors to FMM. It also aims to review current research gaps given the limited current available options of treatment and the scarce evidence regarding FMM. Finally, potential future developments are discussed.

### 1.1. Surgical approaches

The advances in the understanding of neurosurgical anatomy, the development of surgical techniques, and the development of technologies such as neuronavigation systems, real-time angiography, angled endoscopes, artificial intelligence, and augmented reality (11), have improved the surgical results of FMM in terms of morbidity and mortality, which have substantially decreased in the last decades. Tumor encasement of the vertebral and basilar arteries as well as of the lower cranial nerves represents the most important aspect. Detailed surgical planning, as well as the application of microsurgical techniques, are paramount for achieving this goal. For ventral FMM, many approaches have been proposed. Here we present the pros and cons of the most commonly applied procedures.

#### 1.1.1. Transcondylar approach

**Figure 1:**
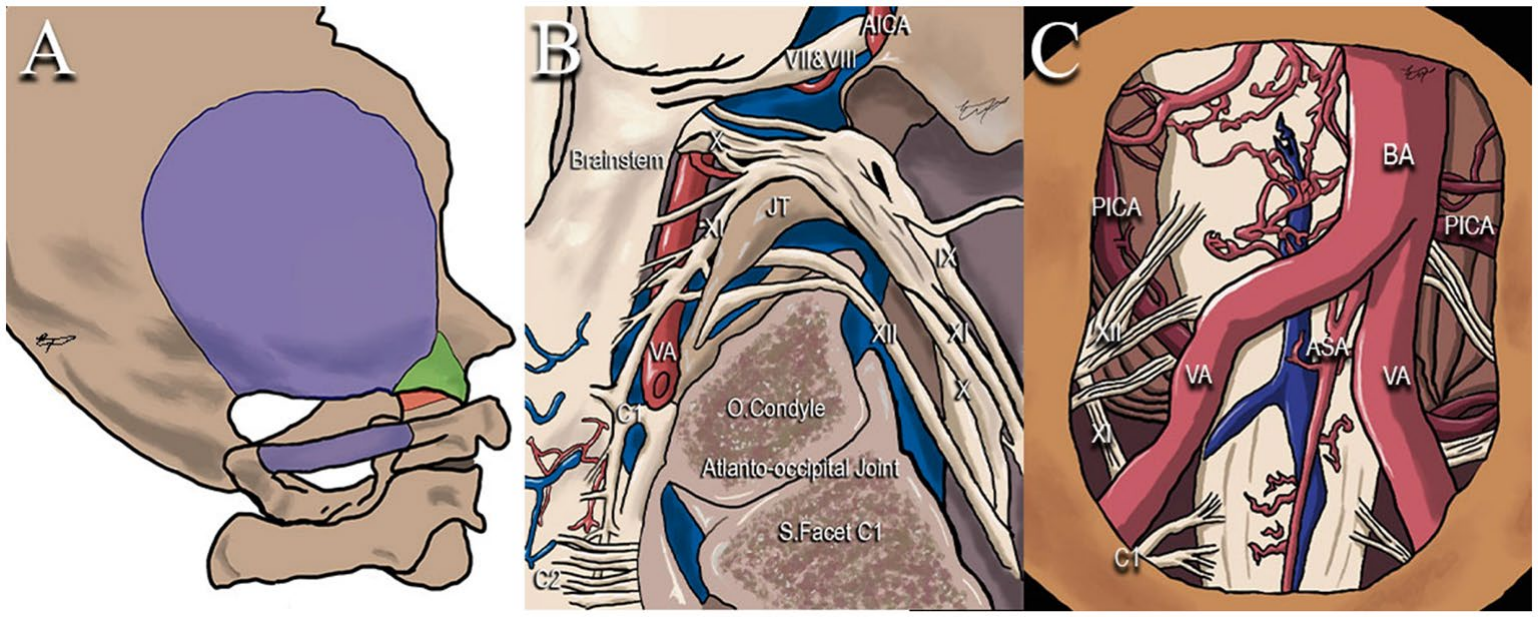
Illustration of surgical approaches to foramen magnum meningiomas. AICA: anterior inferior cerebellar artery. VII&VIII: facial (CN VII), and vestibulocochlear (CN VIII) nerves. IX: glossopharyngeal nerve. X: vagus nerve. XI: accessory nerve. XII: hypoglossal nerve. JT: jugular tubercle. VA: vertebral artery. BA: basilar artery. PICA: posterior inferior cerebellar artery. ASA: anterior spinal artery. C1: first cervical nerve.

Variations of this approach have been described according to the patient’s position, skin incision, muscle reflection, and craniotomy. These variations include the far lateral, occipital transcondylar, atlantooccipital trans articular, supracondylar, trans tubercular, para condylar, and other minimally invasive approaches (12–16). Figure 1 represents some of these variations. The differences among these approaches may influence exposure, mechanical instability, and neurovascular injuries (17). A tailored bone resection among these variations is presented in Table 1. Here we resume some steps of this approach.

**Table 1:** Variations of transcondylar approach.

Approach	Bone structures drilled	Potential benefits and blind spots exposed
Far lateral (posterolateral)	Suboccipital bone and hemilamina of C1	Behind the VA and medial to the occipital and atlantal condyles
Occipital transcondylar	Suboccipital bone, hemilamina of C1, and occipital condyle without entering the hypoglossal canal (the posterior third of the occipital condyle).	If there is a need to complete a circumferential dural incision around the site at which VA penetrates the dura for mobilization. Access to lesions ventral to the artery and in front of the cervicomedullary junction. Adequate for lesions requiring greater anterior and superior exposure.
Atlantooccipital trans articular	Suboccipital bone, hemilamina of C1, occipital condyle without entering the hypoglossal canal (the posterior third of the occipital condyle), and lateral mass of C1 (superior articular facet)	Gain access to extradural lesions located along the anterior and lateral margins of the foramen magnum.
Supracondylar	Suboccipital bone, hemilamina of C1, and occipital condyle and entering the hypoglossal canal (including the jugular tubercule).	Directed above the occipital condyle to the hypoglossal canal to the lateral side of the clivus. Also provides access to the jugular tubercle and inferior petroclival junction
Transtubercular	Suboccipital bone, hemilamina of C1, occipital condyle and entering the hypoglossal canal.	Unblocks access to the area in front of the glossopharyngeal, vagus, and accessory nerves. Also, increases visualization of the area in front of the brainstem and exposes the origin of the posterior inferior cerebellar artery.
Paracondylar	Suboccipital bone, hemilamina of C1, the jugular process (the quadrangular area) lateral to the occipital condyle, and a posterior partial mastoidectomy (occasionally).	Provides excellent exposure on the side of the exposure and extends across the midline to the medial aspect of the contralateral atlantooccipital joint and the lower clivus. Provides access to the posterior part of the jugular foramen and the extratemporal segment of the facial nerve.
Transjugular	Suboccipital bone, hemilamina of C1, the jugular process to the posterior surface of the jugular bulb. Occasionally extended laterally to the jugular foramen into the posterior aspect of the mastoid	To access the mastoid segment of the facial nerve and the stylomastoid foramen
Minimally invasive supracondylar trans tubercular	A small portion of the occipital bone, the posterior aspect of the occipital condyle, and the superior facet joint of C1	It does not require a C1 hemilaminectomy nor extensive exposure to the extracranial VA. It is an ideal companion to endoscope-assisted neurosurgery.
Minimally invasive natural anatomical gaps-posterior cervical	No bone resection is needed.	To reach ventrally located tumors of the CVJ without the need for resecting any bony structures. It decreases musculoskeletal morbidities.

A tailored bone resection as well as the potential benefits and blind spots exposed with each variant are described.

**Figure 2:**
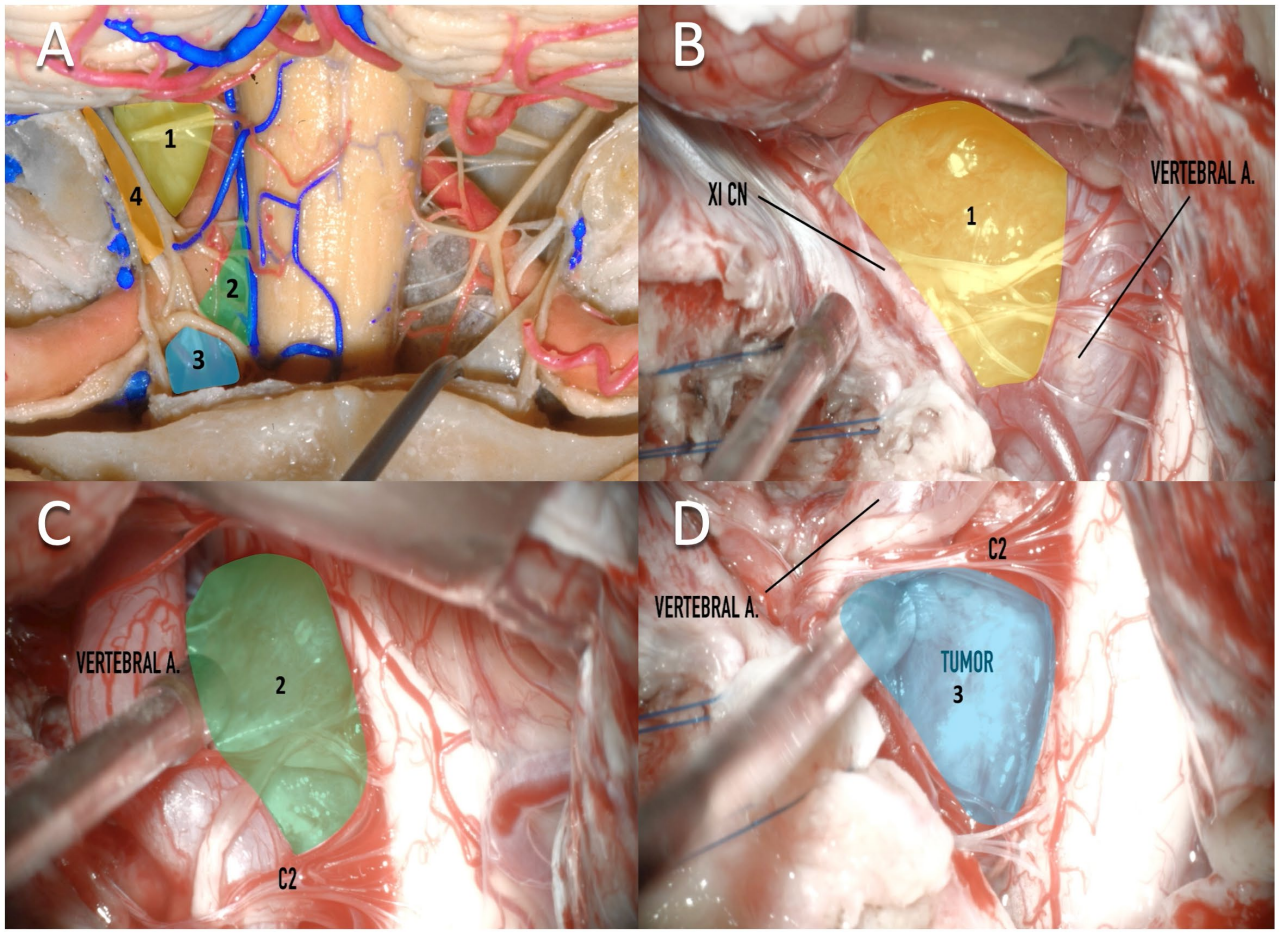
Clockwise classification of surgical corridors for resection of foramen magnum meningiomas. **(A)** A cadaveric dissection demonstrates a posterior exposure of the medulla and structures of the craniovertebral junction. Four corridors are demonstrated which are predominantly created by relationships between the vertebral artery and the XI cranial nerve. **(A,B)** *Corridor 1*: between the XI nerve and the vertebral artery (shown in yellow). **(A,C)** *Corridor 2*: below the vertebral artery, lateral to the medulla, and above the C2 nerve. **(A,D)** *Corridor 3*: Below to C2, medial to the medulla, and lateral to the vertebral artery. **(A)** *Corridor 4*: Lateral to the XI cranial nerve. XI CN, XI cranial nerve; vertebral a, vertebral artery.

In the classic far lateral, there is usually no need to open the foramen transversarium of C1. However, a suboccipital craniotomy with a C1 laminectomy is traditionally performed. The VA can be mobilized after the opening of the foramen transversarium, a maneuver that is helpful in cases when additional ventral exposure is necessary. Drilling of the occipital condyles can be performed, as needed, to maximize dura reflection and ventral exposure in the CVJ (18, 19). This approach allows a direct view of neurovascular structures which facilitates a sharp dissection. The exposure of cranial nerves (CN) IX, X, XI, and XII generate anatomical corridors (vago-accesory triangle, supra-hypoglossal triangle, hypoglossal-hypoglossal triangle, infra-hypoglossal triangle) (20, 21) between them to coagulate and resect the tumor (17). Figure 2 demonstrates a clockwise classification of surgical corridors to FMM. However, the inherent risks of the drilling, as well as of the constant mobilization of the intra and extradural segments of the VA and the lower CNs continue to be a constant challenging task during the procedure. With respect to venous bleeding, applying hemostatic agents and pressure would be sufficient to avoid postoperative bleeding. This is important given the large collateral venous drainage in this area (22, 23). Additionally, this approach may have contralateral blind spots. Expertise and adequate skull base surgery training are mandatory in order to avoid critical neurovascular injuries.

The transcondylar approach allows wide surgical access to the pontomedullary junction, the anterolateral foramen magnum, and to the lower third of the clivus (24). To get enough access, positioning is paramount for this approach. The patient can be placed in a semi-sitting position or in a modified park bench position (24). The semi-sitting position provides a wider angle of view and improves venous return, but the rich net of veins around the cervical muscles and the VA offers the risk of air embolism (25, 26). This risk increase if the condylar emissary vein, sigmoid sinus, and jugular bulb, are to be exposed. This approach provides optimal exposure and provides a working angle anterior to the brainstem and flush with the clivus. The skin incision, as well as the muscle dissection, should be sufficient to expose adequately C1 for drilling and prevent VA injury. These features may impact on the duration of the procedure. This approach gives an ideal dissection in a step-by-step fashion to expose the extra and intracranial VA, as well as all intracranial neurovascular structures. This includes the dissection of the three layers of muscles: (1) the superficial layer: the trapezius and sternocleidomastoid muscles, (2) the middle layer: the splenius capitis, longissimus capitis, semispinalis capitis, and splenius cervicis muscles, and (3) the deep layer: the complete suboccipital triangle, which is made up of the rectus capitis (major and minor), and the superior oblique muscles. This approach provides a step-wise dissection of every anatomical structure, making it easier to identify each structure and mobilize any of them, if necessary, in order to increase the angle of attack.

The need for drilling additional bone is based on the caudal and lateral invasion of the tumor into the hypoglossal canal. Unfortunately, when substantial bone removal is necessary, it will likely destabilize the CVJ. Compared to EEA, the transcondylar approach can provide a wide exposure not only to the tumor, but to almost all neurovascular structures of the posterior fossa, including the CNs V through XII, the basilar artery, VA, the posterior inferior cerebellar artery, and the anterior inferior cerebellar artery (24). Although this approach is generous in terms of anatomical exposure, it is demanding, time taking, and requires advanced skull base training. All variations of the transcondylar approach require a detailed knowledge of the skull base and each of them opens a different blind spot (Table 1), which would improve angles of attack in certain cases.

Minimally invasive approaches have been described, including variations of the transcondylar approach like the minimally invasive supracondylar trans tubercular approach to the lower clivus in cadaveric specimens (14) and a newly fashioned “Natural Anatomical Gaps-Posterior Cervical Approach,” recently described in a small series of four patients (15). The minimally invasive supracondylar transtubercular approach uses a small S-shaped incision to resect a small portion of the occipital bone and drilling the posterior aspect of the occipital condyle and the superior facet joint of C1. This approach may be an alternative that has the potential to minimize operative morbidity and decrease the need for VA manipulation. In addition, with this approach, there is no need for removal or exposure of the C1 arch (14). On the other hand, the Natural Anatomical Gaps-Posterior Cervical approach is focused on saving bone resection given that the posterior aspect of the CVJ exhibits natural bony openings that may be used to access the upper cervical spinal canal, the foramen magnum, and the lower clivus, given the theoretical access supplied by the interspace between the occiput and the posterior arch of C1, C1 and the cranial edge of the lamina of C2, and between the lamina of C2 and the superior edge of the lamina of C3 (15). Unfortunately, this approach is suitable only for those patients with tumors not extending higher than the CVJ and a fairly mobile C-spine with sufficient width of the posterior bony gaps. These approaches are limited in different aspects, especially by the size and location of the tumor, as well as by the tumor consistency, which may require additional exposure and bone resection.

#### 1.1.2. Endoscopic endonasal approach

The EEA continues to develop given the constant advancements in the capacities of the scopes as well as the maneuvering of the instruments through the nasal cavity, the sphenoid sinus, and the nasopharynx (27). EEA allows a straightforward exposure of the meningioma, a circumferential visualization, an intra-tumoral debulking before arachnoid dissection, and early devascularization. However, many limitations have been described: the learning curve for an adequate procedure is usually delayed, and the evident risk of cerebrospinal fluid (CSF) leakage despite a multilayer reconstruction remains the most concerning limitation (28). Additional anatomical boundaries limit the lateral exposure for large tumors and can lead to a partial resection of the tumor. Arachnoid dissection can be performed safely and direct visualization of perforating branches permits a safe anterior decompression. Angled endoscopes as well as improved visualization of structures have improved the extent of resection and have allowed extended versions including drilling the anterior arch of C1 and the odontoid tip for those FMM with more caudal extension (29). In addition, the endoscopic transclival transcondylar variation (also called the far medial approach) allows an improved surgical corridor when compared to the classic transclivus approach, facilitating exposure and resection of the ventral foramen magnum and jugular tubercle meningiomas (27, 29). The straightforward midline approach to the tumor prevents the constant manipulation of the lower CNs as well as the VA. However, the bony and neural structures limit dural tail resection in the most lateral aspect in some selected cases. Other limitations are related to size, caudal extension, vascular encasement, and potential postoperative instability (30–32). The use of advanced closure techniques is mandatory and training for complex multilayer reconstruction is important for satisfactory outcomes (27). Inherent risks including neurovascular injuries are correspondent to the challenging location of FMM and remain the most remarkable limitation for any approach.

The EEA has been increasing in use, given the familiarity of neurosurgeons given dedicated training in advanced endoscopic techniques. The magnification for better exposure has improved visualization in dark and stretched corridors in the skull base from below. Unfortunately, a straightforward approach to ventral FMM is limited by lateral access given the direct obstruction posed primarily by the internal carotid artery, the VA, and CNs (mainly CN XII laterally) (33). This approach is especially useful for midline anterior FMM, when a complete removal of the dura can be performed, or when there is a pure ventral compression of the brainstem. The dissection of the tumor from the pons and the medulla using endonasal techniques requires a high level of experience and adequate microsurgical instrumentation to perform sharp microvascular dissection based on endoscopic visualization and is therefore recommended to be pursued only in centers with an advanced level of experience. The use of microsurgical techniques using the endoscope has improved with better lighting but is limited due to decreased surgical freedom through the nose. Furthermore, such an approach is not ideal for all tumors and those with significant lateral and inferior extension are better managed via transcranial approaches. The addition of lateral extensions, such as the endoscopic endonasal transclival transcondylar approach or endoscopic transclival far medial approach leads to additional lateral exposure while preserving low rates of morbidities and minimizing the risk on craniocervical instability (29).

### 1.2. Surgery versus radiotherapy

Surveillance and conservative management are warranted for small and asymptomatic FMMs. Tumors with progressive enlargement or symptomatic tumors have surgery as a primary treatment option, but adjuvant radiotherapy, including stereotactic radiosurgery (SRS) or fractionated external beam radiotherapy, or a combination of both resection and radiotherapy are alternatives (34). Although GTR is the ideal goal of surgery, this is not always possible for FMMs, and SRS has been proposed also for alternative complementary treatment for residual tumors when reducing mass effect is not necessary (34). The evidence is insufficient to determine if upfront radiosurgery should be recommended in favor of conservative treatment for residual tumors and literature has demonstrated satisfactory results in both scenarios (35, 36). SRS for FMM frequently results in tumor control or tumor regression, as well as symptom improvement (37). However, this treatment should be carefully selected in a case-by-case manner, considering tumor size and volume, compression of the brainstem, previous treatments, and the patient’s neurological status and comorbidities. Mean treatment doses of 12-13 Gy are usually prescribed and have been associated with good results, however, proximity of tumors to the brainstem may limit the ability to deliver the full dose to the entirety of the tumor and lead to suboptimal SRS. Additionally, such a close relationship may expose the brainstem to increase the chances of injury secondary to radiation and the development of new neurological deficits.

### 1.3. Current research gaps

Unfortunately, FMM is not frequent and limits the comparison of treatment strategies. As meningiomas in other locations, FMM is amenable to conservative treatment, surgical resection, and/or adjuvant therapy. New strategies including new surgical corridors, as well as defining consensus of treatment protocols are necessary in order to offer a less morbid treatment for these patients. Further molecular profiling and genetic analysis as well as other treatment modalities including immunotherapy, and CAR T cells, among others (38), remain inconclusive and are amenable to further comprehensive investigation.

### 1.4. Potential future developments

Regarding surgical treatment, the debate to choose the best surgical approach will continue as each tumor always present different anatomical relationships. Despite this tumor being infrequent, the constantly developing surgical study of this anatomy in cadaveric specimens will guide us to a better path. In addition, long-term follow-up would be helpful to determine the risk of recurrence as well as the need for further treatment. Prognostic markers of tumor recurrence are needed and will guide neurosurgeons to define a better strategy for those selected cases.

On the other hand, improved diagnostic and prognostic markers are necessary to provide new targeted drug treatments. In the years to come the advances in molecular profiling and immunophenotyping of meningiomas may lead to the development of new personalized therapeutic strategies. FMMs are part of a large group of posterior fossa meningiomas, that have a specific molecular profile (39), however, tumor tissue analysis is scarce and would complement information to develop new targeted therapies.

## 2. Discussion

There is no consensus on the optimal management of purely ventral FMMs. The rare presentation of these tumors and the neurovascular relationships establish a difficult decision to make. The approach selection as well as the complementary treatment if necessary are controversial. The comprehensive study of neuroanatomy is fundamental to understanding the complex relationships of the tumor as well as the intraoperative strategies to perform the maximal safe resection (2, 4, 17). All tumors should have a tailored approach that would minimize morbidity and achieve the highest possible resection (31). Ventral FMM are the most challenging ones and additional investigation on surgical approaches and adjuvant treatments is necessary. In conclusion, GTR of FMM remains the gold standard of treatment and should be performed whenever possible. Far lateral transcondylar approach is the most commonly applied surgical approach for the resection of anterior and lateral FMM; EEA represents a relatively new surgical option and can lead to successful results in the treatment of anterior FMM when performed in centers with high levels of experience in endoscopic skull base surgery. Fractionated radiation therapy and radiosurgery represent an option for certain cases where the tumor is of small dimensions or for the management of residual tumors or recurrent tumors where neurovascular decompression is not a goal in the treatment strategy. Advances in molecular profiling, as well as genetic analysis, may guide further treatment. Current information regarding FMM is scarce and further investigation into these controversies and research gaps is still necessary.

## Author contributions

MB, SL, JA, WC-L, EL, EO-R, and AC: conceptualization, formal analysis, investigation, methodology, validation, and writing—review and editing. EO-R: project administration. AC: supervision. MB, EO-R, and JA: roles and writing—original draft. All authors contributed to the article and approved the submitted version.

## Conflict of interest

The authors declare that the research was conducted in the absence of any commercial or financial relationships that could be construed as a potential conflict of interest.

## Publisher’s note

All claims expressed in this article are solely those of the authors and do not necessarily represent those of their affiliated organizations, or those of the publisher, the editors and the reviewers. Any product that may be evaluated in this article, or claim that may be made by its manufacturer, is not guaranteed or endorsed by the publisher.

## Abbreviations

CN: cranial nerve
CSF: cerebrospinal fluid
SRS: stereotactic radiosurgery
CVJ: craniovertebral junction
EEA: endoscopic endonasal approach
FMM: foramen magnum meningioma
VA: vertebral artery
